# Supplementary material for: Long noncoding RNA LncHIFCAR/MIR31HG is a HIF-1α co-activator driving oral cancer progression
Source: Nat Commun. 2017 Jun 22;8:15874. doi: 10.1038/ncomms15874 (PMC5489688; doi:10.1038/ncomms15874)
Supplement: Supplementary Information — Supplementary Figures and Supplementary Tables [file ncomms15874-s1.pdf]

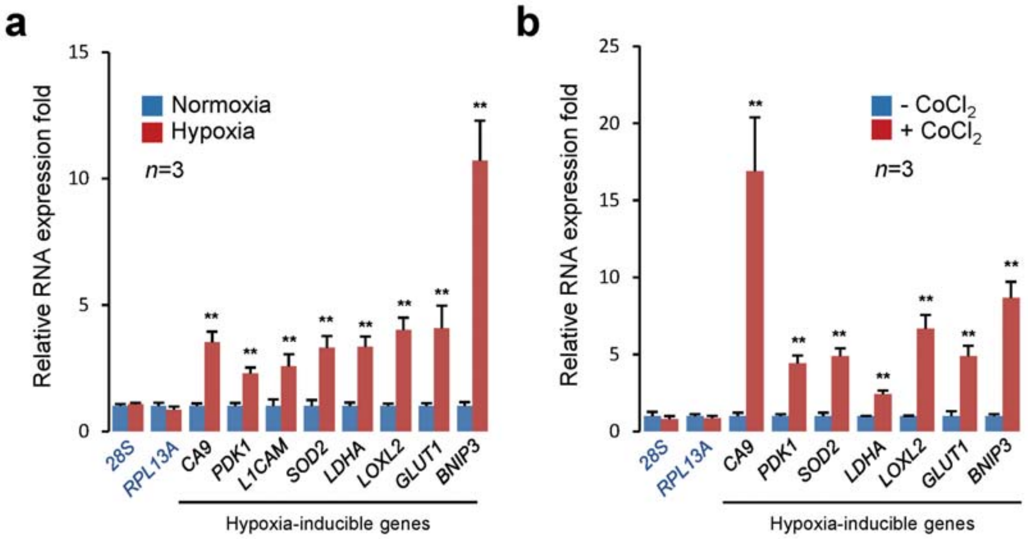

Supplementary Figure 1. Validation of a gene set known to be relevant for hypoxia treatment confirmed the robustness of the initial screening. Quantitative reverse transcriptase–PCR (qRT–PCR) analysis of hypoxia-inducible genes in HeLa cells under hypoxia (1% O_2 for 16 hours; **a**) or treated with hypoxia-mimetic agent cobalt chloride (100 μM for 16 hours; **b**) relative to untreated cells. Data were normalized to 18S rRNA level whereas 28S rRNA and ribosomal protein L13a (*RPL13A*) serves as hypoxia-stable housekeeping gene controls. Results are presented as mean ± SD. *n*, the number of independent experiments performed; Student's *t* test, *P<0.05; **P<0.01.

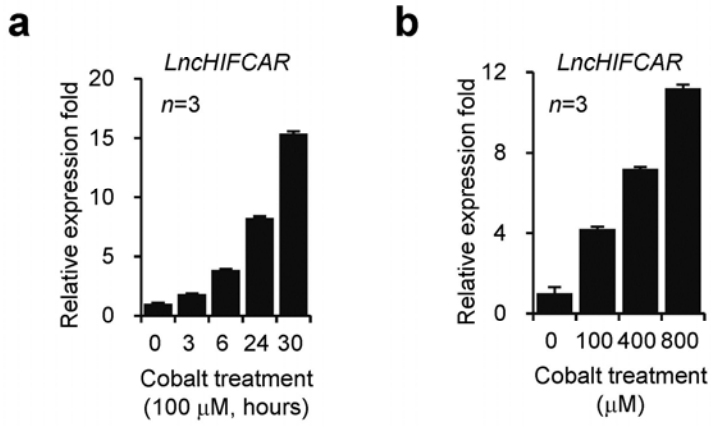

Supplementary Figure 2. *LncHIFCAR* is up-regulated by chemical induced pseudo-hypoxia in a time- and dose-dependent manner. HeLa cells were treated with 100 μM $CoCl_2$ for different time period (**a**) or with the various doses for 6 hours (**b**) as indicated. Total RNA was then isolated and subjected to quantitative real-time PCR analysis normalized to *RPLP0*. Results are presented as mean ± SD. *n*, the number of independent experiments performed.

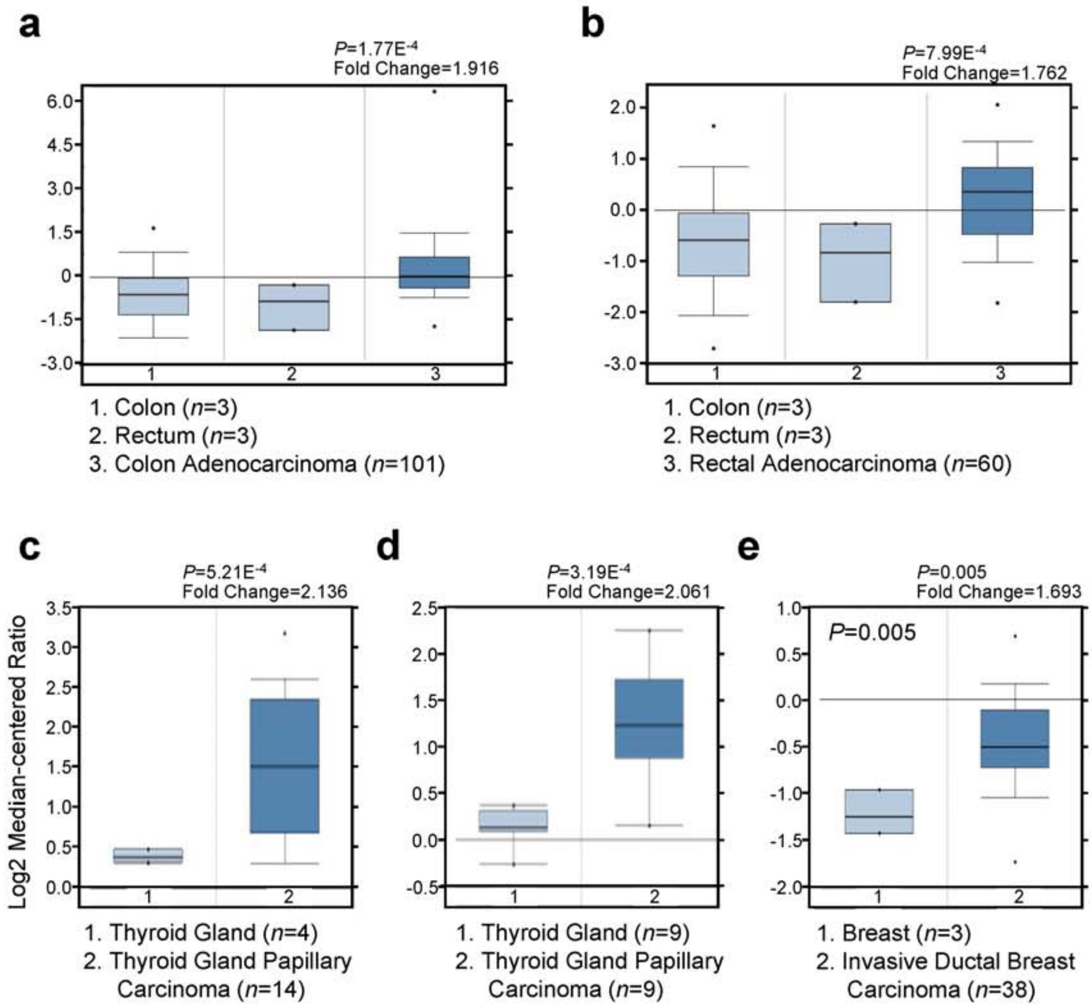

Supplementary Figure 3. *LncHIFCAR* lncRNA is highly up-regulated in human tumors. Oncomine (www.oncomine.org) boxed plot showing elevated *LncHIFCAR* (search term: *LOC554202*) expression levels in different types of human cancer tissues. *LncHIFCAR* expression levels extracted in TCGA colorectal (**a**,**b**), He thyroid (GEO accession number GSE3467; **c**), Vasko thyroid (GEO accession number GSE6004; **d**), and Zhao breast (GEO accession number GSE3971; **e**) datasets are presented as box-plot diagrams, with the box encompassing 25th–75th percentile. Solid horizontal black line represents the median while error bars indicate the 10th to 90th percentile. Statistical analyses between different patient groups were examined by Student's *t* test.

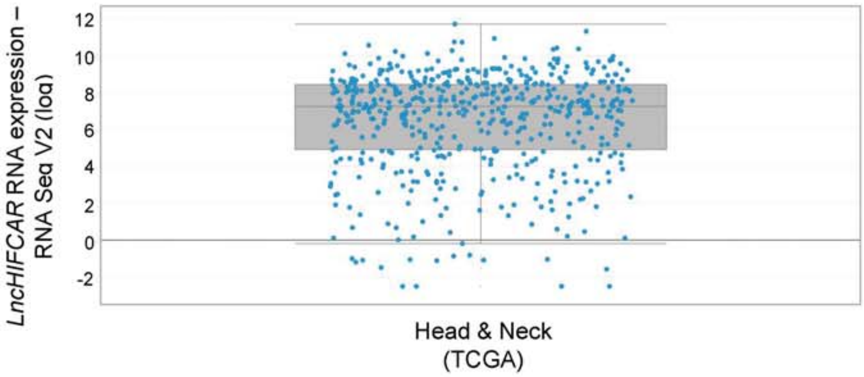

Supplementary Figure 4. *LncHIFCAR* is highly expressed in TCGA head and neck squamous cell carcinoma collectives. Expression levels of *LncHIFCAR* were analyzed with the head and neck squamous cell carcinoma dataset (TCGA, Provisional) using cBioPortal platform (http://www.cbioportal.org). The relative expression of *LncHIFCAR* to its expression distribution in all profiled tumor samples is shown.

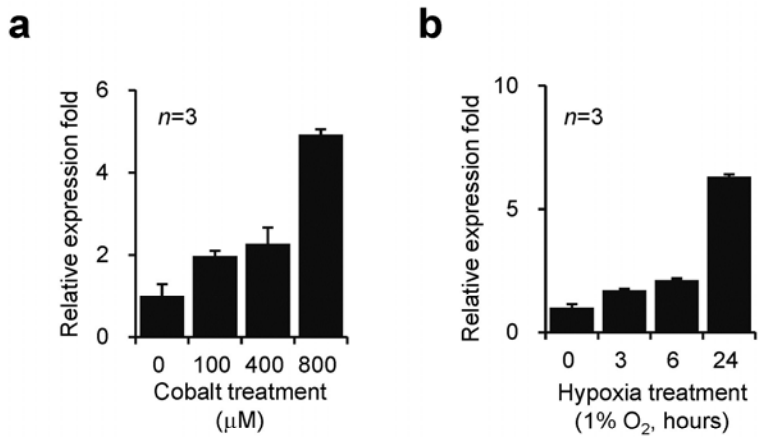

Supplementary Figure 5. *LncHIFCAR* is highly up-regulated in SAS cells upon chemical-induced pseudohypoxia or physical hypoxia. Human oral cancer SAS cells subjected to the following treatments were harvested. Total RNA was then isolated and subjected to real-time RT-PCR analysis for the expression level of *LncHIFCAR* normalized to the expression of *RPLP0*. (**a**) The expression level of *LncHIFCAR* in SAS cells was induced after $CoCl_2$ treatment for 16 hours in a dose-dependent manner. (**b**) The expression level of *LncHIFCAR* in SAS cells was induced in a time-dependent manner after hypoxia treatment for 24 hours. Results are presented as mean ± SD. *n*, the number of independent experiments.

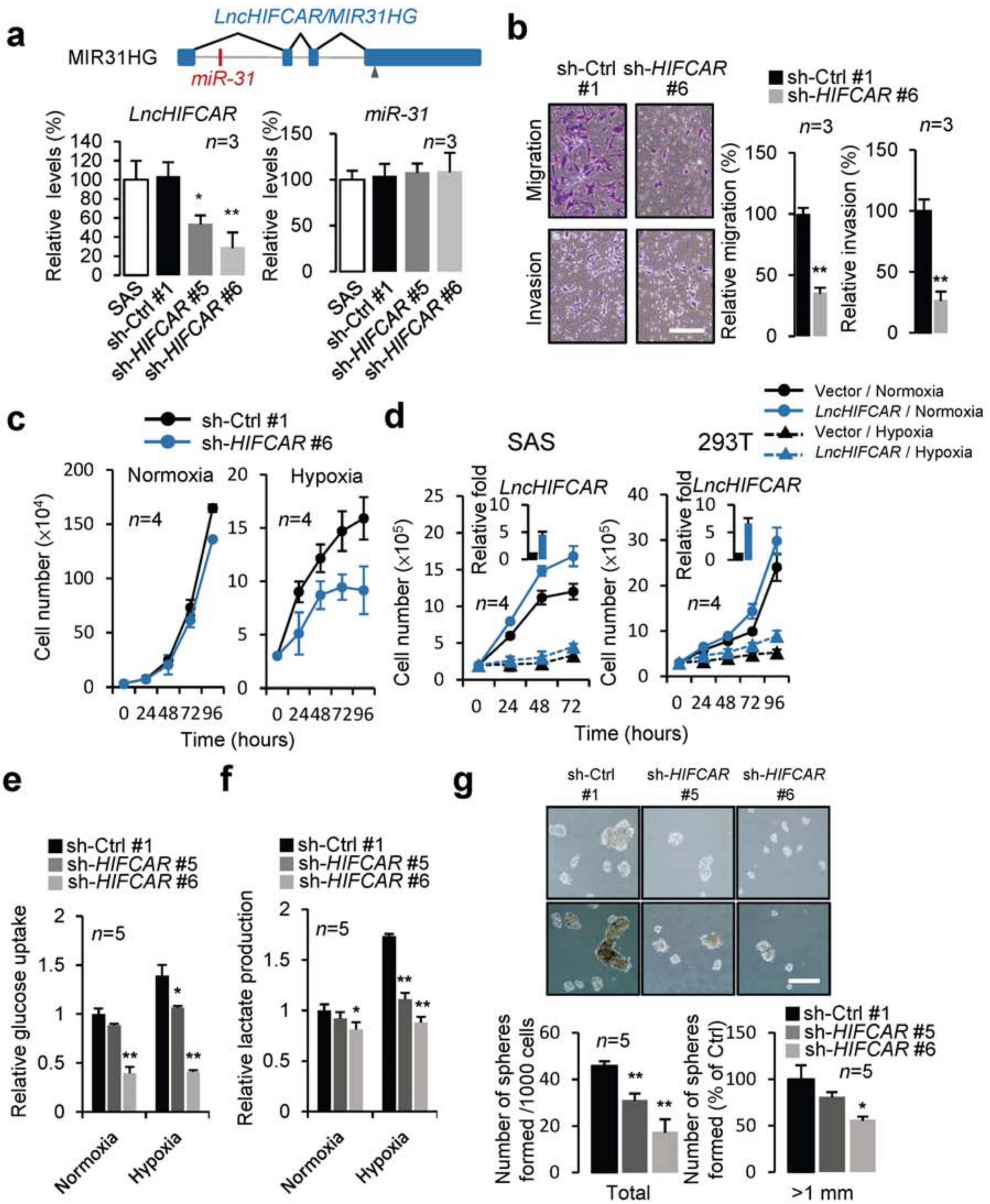
a
LncHIFCAR/MIR31HG
MIR31HG
miR-31
LncHIFCAR
miR-31
Relative levels (%)
n=3
SAS
sh-Ctrl #1
sh-HIFCAR #5
sh-HIFCAR #6
b
sh-Ctrl #1
sh-HIFCAR #6
Migration
Invasion
Relative migration (%)
Relative invasion (%)
c
Normoxia
Hypoxia
n=4
Cell number (×10^4)
Time (hours)
d
Vector / Normoxia
LncHIFCAR / Normoxia
Vector / Hypoxia
LncHIFCAR / Hypoxia
SAS
293T
Relative fold
Cell number (×10^5)
e
sh-Ctrl #1
sh-HIFCAR #5
sh-HIFCAR #6
n=5
Relative glucose uptake
Normoxia
Hypoxia
f
Relative lactate production
g
Number of spheres formed /1000 cells
Number of spheres formed (% of Ctrl)
Total
>1 mm

Supplementary Figure 6. *LncHIFCAR*-knockdown impairs tumor sphere formation, glycolytic metabolism, invasion and migration ability of human oral cancer SAS cells. (**a**) Establishment of *LncHIFCAR*-knockdown SAS clones. Upper panel: schematic representation of the genomic structure of *LncHIFCAR/MIR31HG*. The targeting site of the designed shRNA is indicated by gray arrowhead. *LncHIFCAR/MIR31HG* is composed of four exons (filled blue boxes), and the miR-31, located within intron 1 is shown with a red horizontal bar. Lower panel: *LncHIFCAR* and miR-31 expression levels in SAS parental (SAS), control vector-expressing (sh-Ctrl #1) and *LncHIFCAR* knockdown (sh-*HIFCAR* #5 and #6) cells were analyzed by quantitative PCR and normalized to *GAPDH* and *U6* level, respectively. (**b**) *LncHIFCAR* promotes oral cancer cell invasion and migration. Transwell invasion and migration assays of sh-*HIFCAR* and control SAS cells were performed in hypoxia. Representative photos and quantitative analysis are shown. (Scale bar, 100 μm) (**c**) Cell growth curve of the control (sh-Ctrl #1) and *LncHIFCAR* knockdown (sh-*HIFCAR* #6) SAS cells under normoxia or hypoxia. (**d**) *LncHIFCAR* overexpression promotes cell growth under normoxia and hypoxia.**.** SAS and 293T cells were transfected with vector or *LncHIFCAR*-expressing plasmids for 24 hours, followed by qRT-PCR analysis of the *LncHIFCAR* levels as shown in the inset. The transfected cells were then cultured in normoxia or hypoxia and counted at indicated time points. Results are presented as mean ± SD. *n*, the number of independent experiments performed. (**e,f**) Knockdown of *LncHIFCAR* reduces hypoxia-induced glucose uptake (**e**) and lactate production (**f**). The glucose and lactate levels in the culture media of the control and *LncHIFCAR*-knockdown SAS cells were measured after 16 hours of normoxia or hypoxia treatment. The data is presented as fold difference compared with the sh-Ctrl level in normoxia. (**g**) Knockdown of *LncHIFCAR* reduces the sphere forming ability of SAS cells. The sh-Ctrl and sh-*HIFCAR* SAS cells were grown in suspension culture to form sphere. Representative phase-contrast microscopic images of the cell aggregates are displayed (Scale bar, 300 μm). Bar graph represents the total number of spheres or the relative number of spheres with diameter >1 mm. Graphs show mean ± SD. *n*, the number of independent experiments performed; Student's *t* test, **P*<0.05; ***P*<0.01; ****P*<0.001.

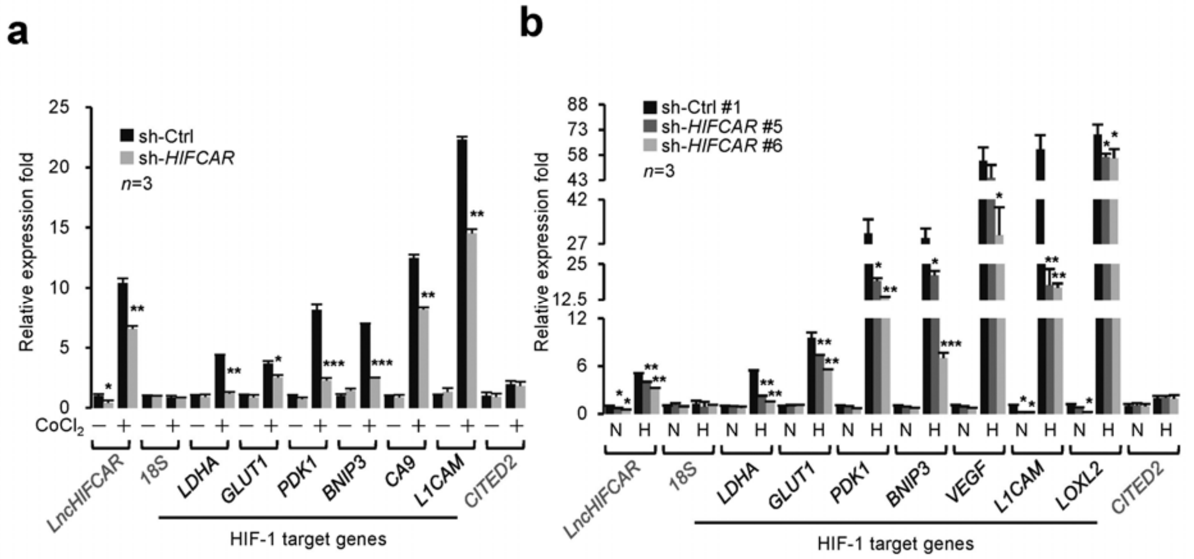

Supplementary Figure 7. *LncHIFCAR* is required for the hypoxia-induced activation of HIF-1 target genes. (**a**) HIF-1 target genes expression in HeLa cells transfected with empty vector (sh-CTRL) or plasmid encoding *LncHIFCAR*-shRNA (sh-*HIFCAR*). 24 hours after transfection, the cells were treated by $CoCl_2$ for 16 hours, followed by qRT-PCR analysis with normalization against *RPLP0* level.(**b**) qRT-PCR analysis of HIF-1 target genes expression (normalized to *RPLP0*) in vector control and *LncHIFCAR*-knockdown SAS clones under 24 hours treatment of normoxia or hypoxia. Graphs show mean ± SD. *n*, the number of independent experiments performed; Student's *t* test, *P<0.05; **P<0.01; ***P<0.001.

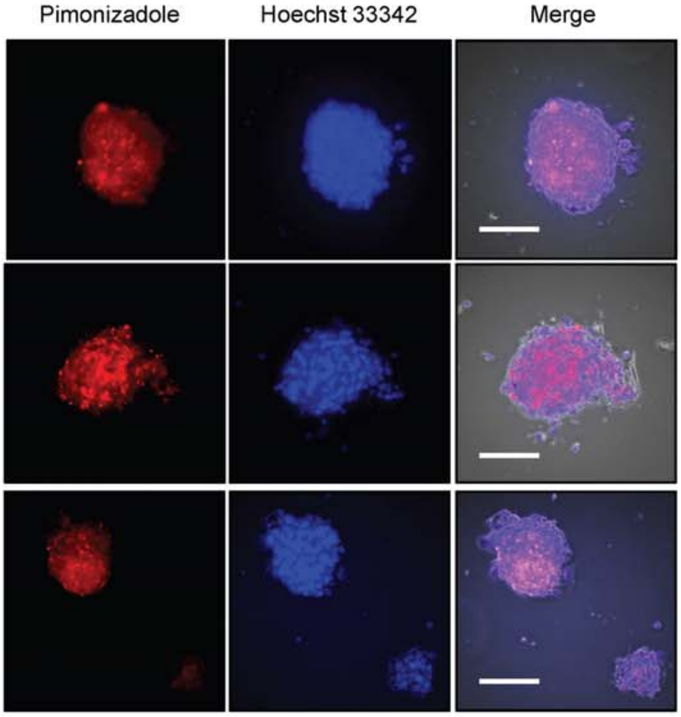

Supplementary Figure 8. Cellular hypoxia developed at the core of spheres visualized by pimonidazole staining. Tumor spheres derived from SAS oral cancer cell were grown in serum-free medium under normoxic conditions and allowed to attach to 0.1% gelatin-coated cover slips for 12 hours. Intracellular pimonidazole complexes indicative of hypoxic conditions were detected by immunofluorescence microscopy using an anti-pimonidazole monoclonal antibody (Hypoxyprobe™-1 Kit, Hypoxyprobe, Burlington, USA). Pimonidazole was applied to the spheres for 1 hour under normoxia. Cell nuclei were visualized by Hoechst staining and bright field is also shown in the merged images (Scale bar, 200 μm).

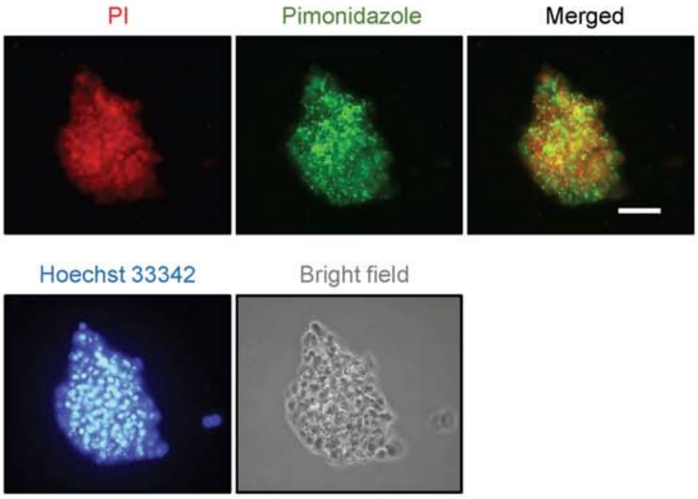

Supplementary Figure 9. The extent of cellular hypoxia and necrosis at the core of the spheres was accessed by fluorescence staining. Tumor spheres derived from SAS oral cancer cell were grown in serum-free medium under normoxic conditions and then tested for cellular hypoxia developed at the core of spheres by applying pimonidazole for 1 hour. Spheres were immunostained for Intracellular pimonidazole complexes indicative of hypoxic conditions. Propidium iodide (PI) fluorescence staining indicated necrotic cells and nuclei were visualized by Hoechst staining (Scale bar, 100 μm).

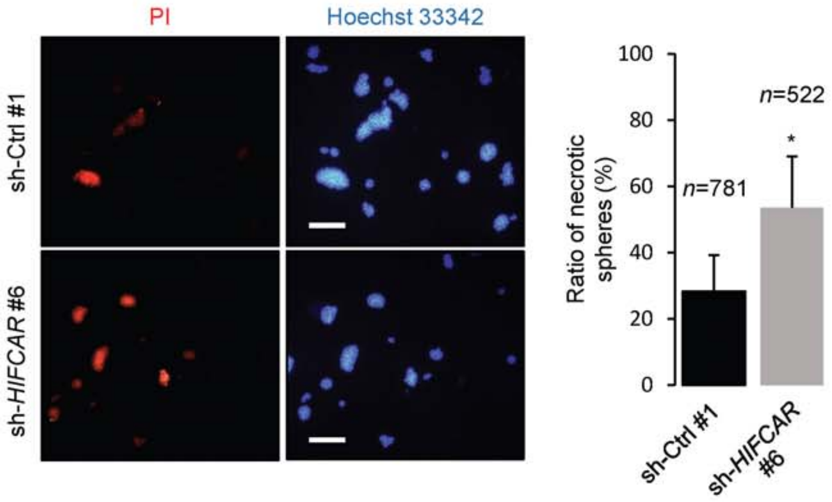

Supplementary Figure 10. Detection of necrosis using propidium iodide (PI) staining for tumor spheres derived from vector control or *LncHIFCAR* knockdown SAS cell lines. Tumor spheres derived from vector control or *LncHIFCAR* knockdown SAS cell lines were grown in serum-free medium under normoxic conditions and tested for necrosis by propidium iodide (PI) fluorescence staining. Spheres of comparable size (50-200 μm) were collected and analyzed by fluorescence microscopy. Nuclei were visualized by Hoechst staining. Three independent experiments were repeated and the representative figures were shown (Scale bar, 200 μm). More than 35 images in each group were randomly selected for analysis and the relative ratio of necrotic spheres were shown. *n*, number of total spheres analyzed. Graphs show mean ± SD. Student's *t* test, *$P<0.05$

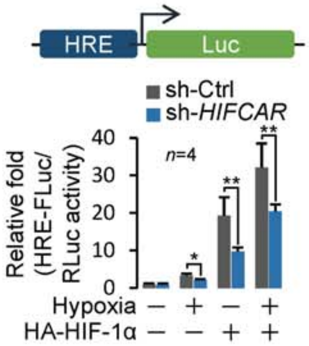

Supplementary Figure 11. *LncHIFCAR* is required for the HIF-1–dependent transactivation. Reporter assay performed in HeLa cells co-transfected with the indicated plasmids for 24 hours, followed by 24-hour treatment of hypoxia or normoxia. Graphs show mean ± SD. *n*, the number of independent experiments performed; Student's *t* test, *P<0.05; **P<0.01; ***P<0.001.

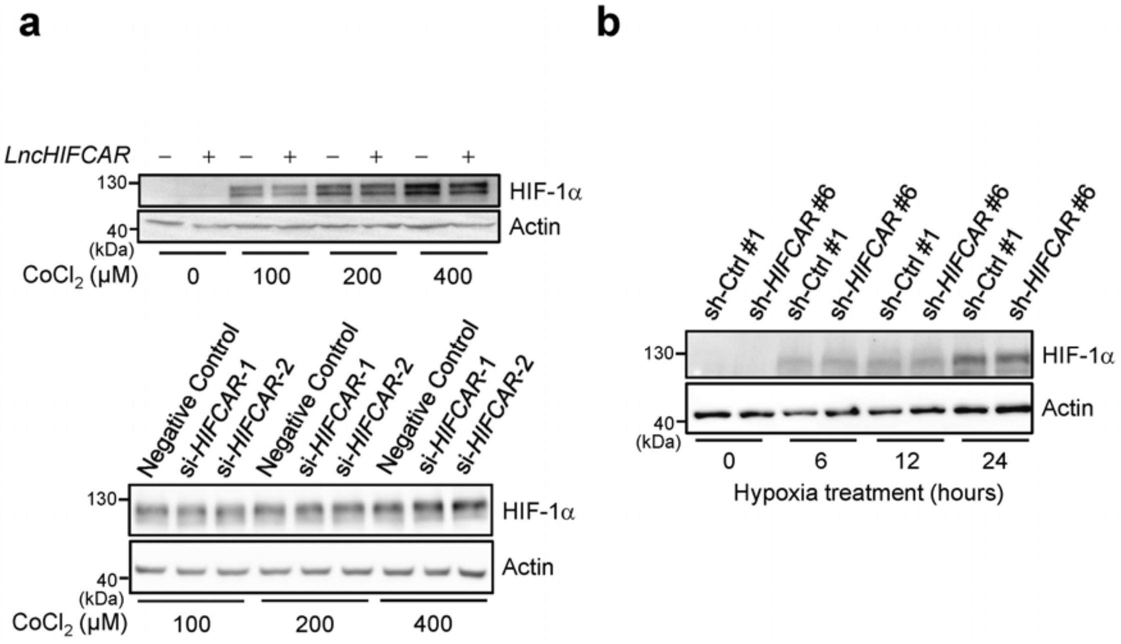

Supplementary Figure 12. The effect of *LncHIFCAR* on HIF-1α protein levels under chemial-induced pseudohypoxia and physical hypoxia. (**a**) Representative (*n*=3) immunoblot analysis of HIF-1α protein levels in control, *LncHIFCAR* overexpressing (upper panel), or *LncHIFCAR* knockdown (lower panel) HeLa cells under $CoCl_2$ treatment with the indicated concentration for 24 hours. To overexpress or knockdown *LncHIFCAR,* HeLa cells were transfected with *LncHIFCAR*-expressing plasmids or siRNAs targeting *LncHIFCAR*- (si-*HIFCAR*) for 24 hours before $CoCl_2$ treatment, respectively. (**b**) Representative (*n*=3) immunoblot analysis of HIF1α protein levels in vector control and *LncHIFCAR* knockdown SAS cell lines under hypoxia treatment for the indicated period of time.

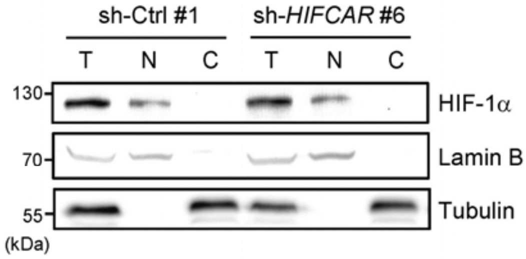

Supplementary Figure 13. The effect of *LncHIFCAR* on HIF-1α translocation under hypoxia. Representative (n=3) immunoblot analysis comparing subcellular fractionation of vector control and *LncHIFCAR* knockdown SAS cell lines after 24 hours hypoxia treatment. Equal amounts of whole cell lysate (T), cytoplasmic (C) and nuclear (N) protein fractions were analyzed by western blotting with HIF-1α antibody. Tubulin and Lamin B are used as loading controls for the cytoplasmic and nuclear fractions, respectively.

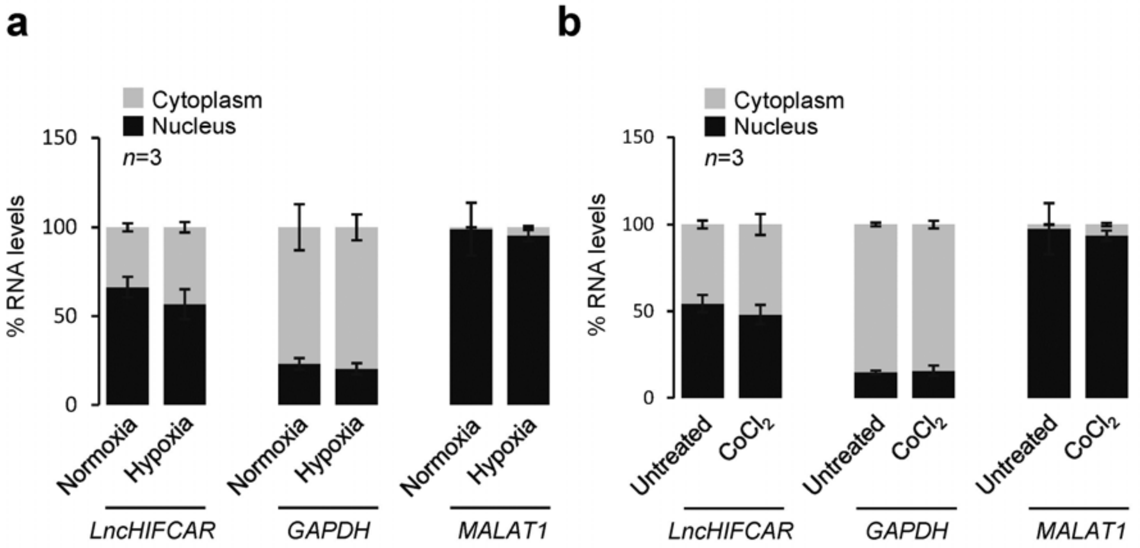

Supplementary Figure 14. The subcellular localization of the *LncHIFCAR* lncRNA in normoxic and hypoxic conditions. Relative ratio of nuclear and cytoplasmic RNA levels of *LncHIFCAR*, *GAPDH* and *MALAT1* measured by qRT-PCR after subcellular fractionation in SAS cells cultured in normoxia and hypoxia (1% O_2 for 16 hours; **a**) or treated with with hypoxia-mimetic agent cobalt chloride (100 μM for 16 hours; **b**). *n*, the number of independent experiments performed. Results are presented as mean ± SD.

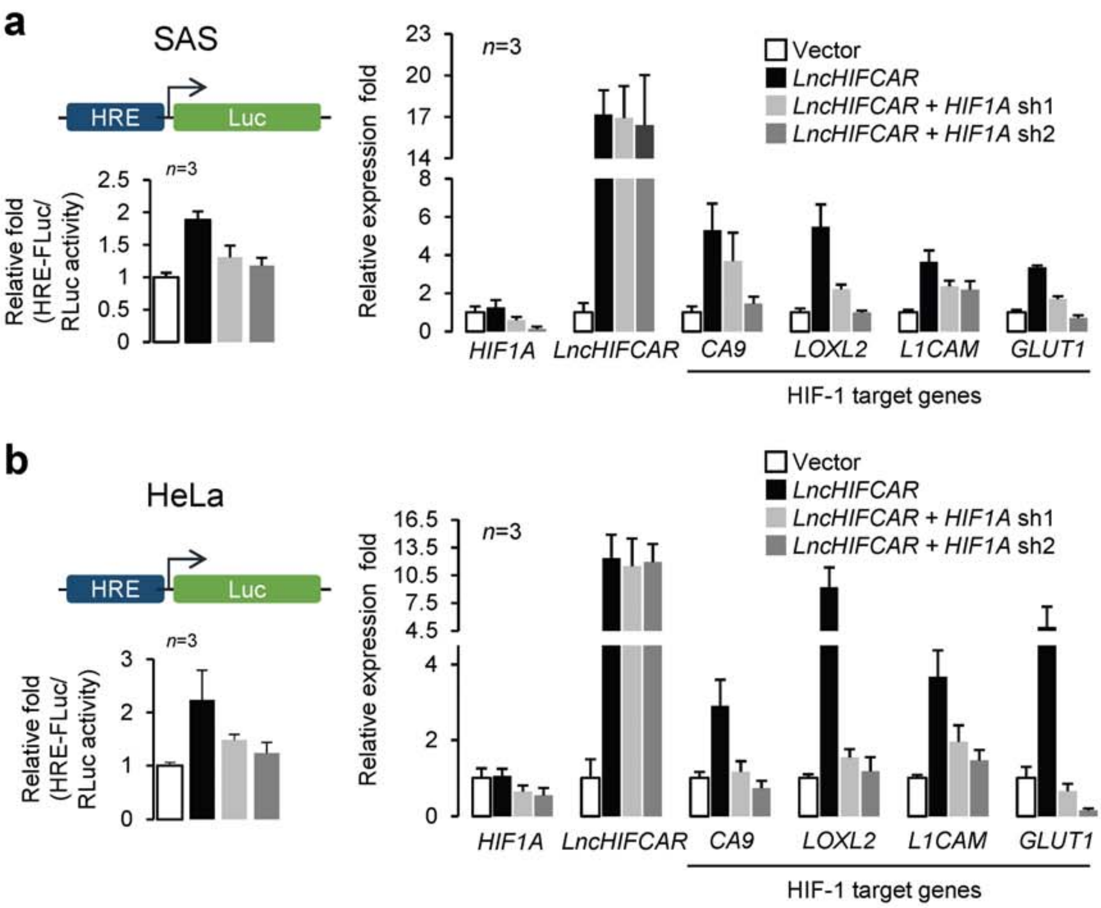

Supplementary Figure 15. HIF-1α is required for *LncHIFCAR*-induced transactivation of HIF-1 target genes under non-hypoxic conditions. The HIF-1α responsive luciferase reporter plasmid was co-transfected with empty vector, plasmids encoding *HIF1A* shRNA (*HIF1A*-sh1 or *HIF1A*-sh2) and *LncHIFCAR* as indicated into normoxic SAS (**a**) or HeLa (**b**) cells for the reporter assay. The RNA expression levels of *HIF1A*, *LncHIFCAR* and HIF-1 target genes were analyzed by qRT-PCR normalized to 18S rRNA levels. Results are presented as mean ± SD. *n*, the number of independent experiments performed.

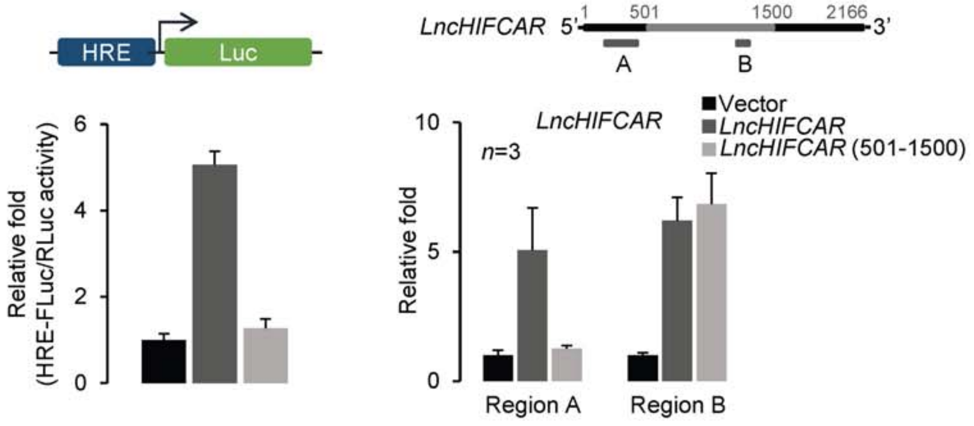

Supplementary Figure 16. HIF1α-binding is essential for *LncHIFCAR*-mediated HIF-1 transactivation. The HIF-1 responsive luciferase reporter plasmid was co-transfected with empty vector, plasmids encoding wild-type or HIF-1α-binding deficient mutant (5000-1500) *LncHIFCAR* RNA as indicated into normoxic HeLa cells for the reporter assay. Specific primers are designed for the quantification of wild-type (A), or mutant (5000-1500) (B) *LncHIFCAR* levels. The positions of the amplified target regions are shown in the schematic diagram, and the primer sequences are provided in Supplementary Data 2. *n*, the number of independent experiments performed. Results are presented as mean ± SD.

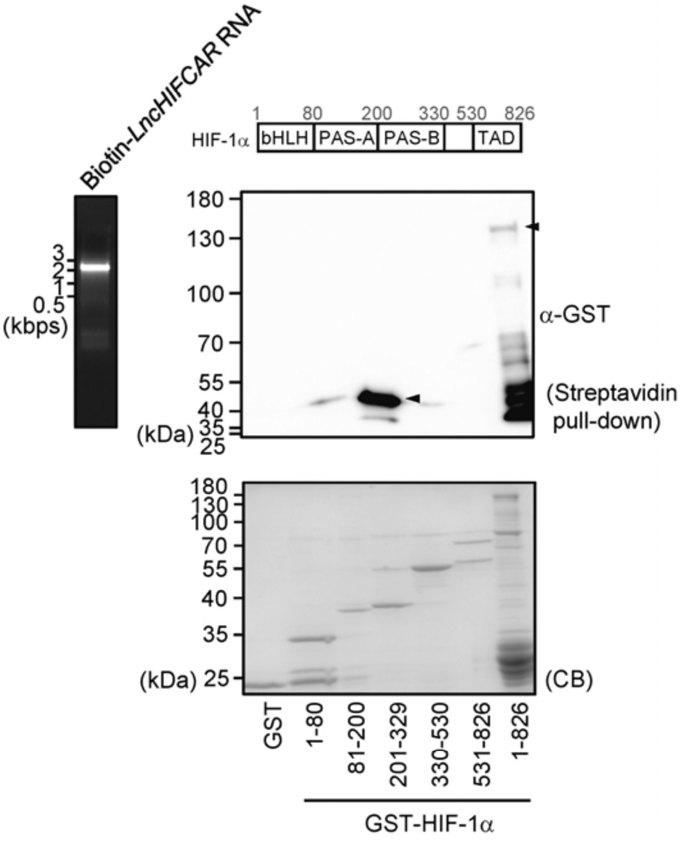

Supplementary Figure 17. Mapping the *LncHIFCAR* binding domain within HIF-1α. Schematic representation of HIF-1α functional domains and GST-HIF-1α variants are shown at the top. Coomassie Brilliant Blue staining (CB) of the purified GST fusion proteins is shown at the bottom. Equal amount of biotinylated *LncHIFCAR* RNAs were incubated with purified GST-fusion proteins and then pulled down by streptavidin beads, followed by western blotting with anti-GST antibody. Representative (*n*=3) immunoblot detection of GST-tagged HIF-1α truncated fusion proteins retrieved by biotinylated *LncHIFCAR* RNA pull-down as indicated by arrowhead was shown. bHLH, basic helixloop-helix; PAS, Per-ARNT-Sim; TAD, transactivation domain.

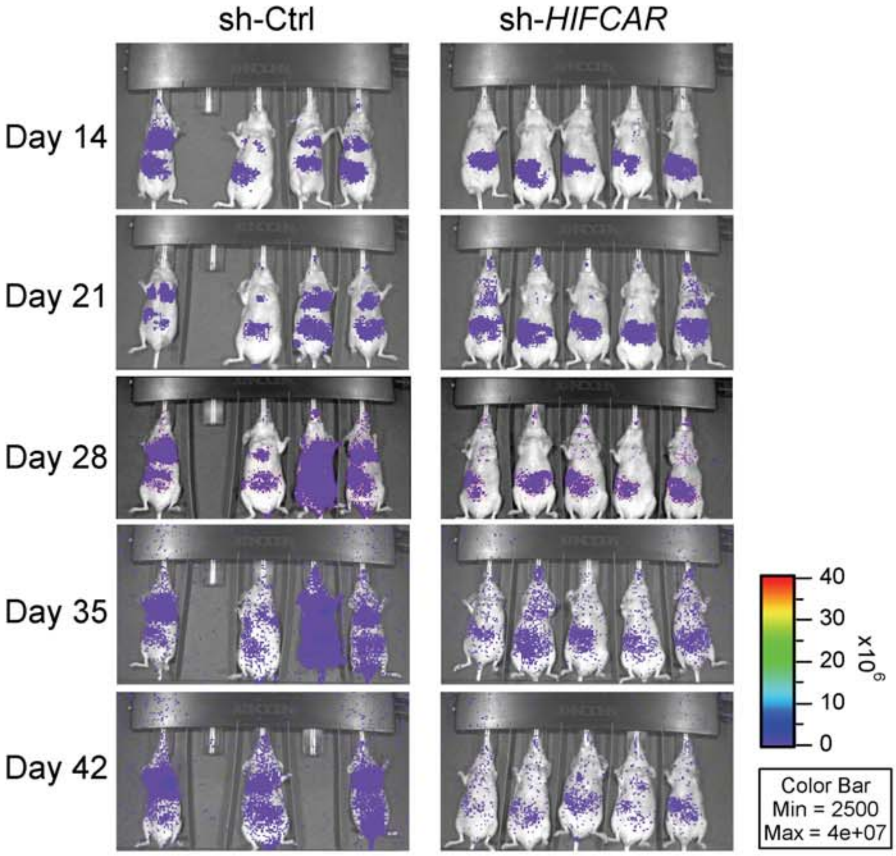

Supplementary Figure 18. *LncHIFCAR* knockdown impairs oral cancer cell metastasis *in vivo*. To investigate experimental lung metastasis, human oral SAS cells expressing control shRNA (sh-Ctrl) or shRNA targeting *LncHIFCAR* (sh-*HIFCAR*) were injected into the tail vein of each anesthetized nude mouse (n=10 in each group). The lung metastases were monitored every week after xenografting using the IVIS Lumina LT series III system. Representative non-invasive bioluminescence images of mice in each group at the indicated times after tail vein injection is shown.

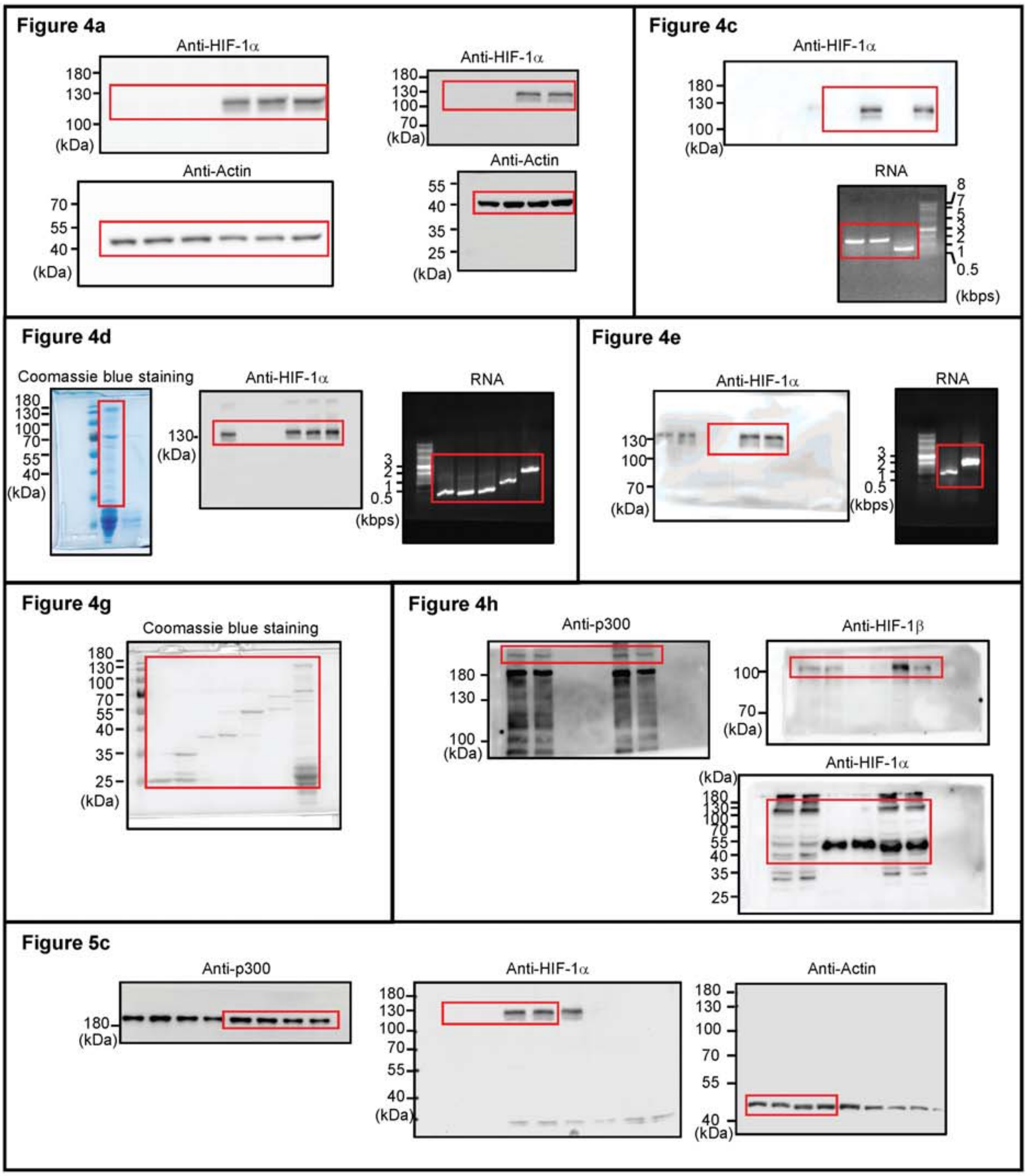

Supplementary Figure 19. Uncut blots and gels. The red boxed regions are presented in the indicated figures in the manuscript.

Supplementary Table 1. The relationship between *LncHIFCAR* expression and clinicopathologic parameters in OSCC

Clinicopathologic parameters	*LncHIFCAR* expression		Number of cases	*P*-value
	High	Low		
**Age (years)**				
≤50	6	14	20	0.037*
>50	14	8	22	
**T status (tumor size)**				
T1+T2 (≤4 cm)	14	15	29	1
T3+T4 (>4 cm)	6	7	13	
**Stage**				
I+II	10	10	20	0.506
III+IV	10	12	22	
**N status (lymph node metastasis)**				
Yes (N1-3)	9	9	18	1
No (N0)	11	13	24	
**Tumor differentiation**				
Well differentiated (G1)	8	18	26	0.01*
Moderately (G2) to poorly differentiated (G3)	12	4	16	

Patients were staged in accordance with the 7th Edition of the AJCC Cancer's' TNM Classification. Quantitative real-time PCR was used to analyze *LncHIFCAR* lncRNA expression in 42 OSCC tissues. The cutoff value of high expression of *LncHIFCAR* was defined as ΔCT ≤ 2.54 to yield maximum sum of sensitivity and specificity for recurrence-free survival analysis. Fisher's exact test was used to determine the correlation between *LncHIFCAR* expression and clinicopathologic parameters. Statistically significant (*P<0.05).

Supplementary Table 2. List of siRNA sequences

Probe name	Sequence (5' to 3')	Reference
**Negative Control (NC)**	Sense: UUCUCCGAACGUGUCACGUTT	
	Antisense: ACGUGACACGUUCGGAGAATT	
**si-*HIFCAR*-1**	Sense: GGGUUUCUGUAUUCAGUUATT	*Oncogene* **35**, 3647-3657 (2016)
	Antisense: UAACUGAAUACAGAAACCCTT	
**si-*HIFCAR*-2**	Sense: CCAGCUGCUGAUGACGUAATT	
	Antisense: UUACGUCAUCAGCAGCUGGTT	

Supplementary Table 3. Specific primers designed for cloning

Primer name	Sequence (5' to 3')	RE	Location*
***LncHIFCAR*-F**	F: AAATTTGAATTCAGGTTCCACGTCCGGCGCCTGGAGAAGGAAGACGCGCG	EcoRI	1-38
***LncHIFCAR*-R**	R: TTTAAAGAATTCGACAAAGGTCACAAAGGATTTATTGTTTTGGCAACAAA	EcoRI	2111-2148
***LncHIFCAR*-F1**	F: ATCGATGAATTCAGGTTCCACGTCCGGCGCCTGGAG	EcoRI	1-24
***LncHIFCAR*-R500**	R: ATCGATGAATTCGAATTCAGGAGGTATTTCCAGAA	EcoRI	486-500
***LncHIFCAR*-F501**	F: ATCGATGAATTCCAAGGCCAGTGTAGAGCC	EcoRI	501-518
***LncHIFCAR*-R1000**	R: ATCGATGAATTCTAGAACCTAGGATCCAAG	EcoRI	983-1000
***LncHIFCAR*-F1001**	F: ATCGATGAATTCTGAAGACATTCTATCTAT	EcoRI	1001-1018
***LncHIFCAR*-R1500**	R: ATCGATGAATTCGTTTACTGGGAATATTGA	EcoRI	1483-1500
***LncHIFCAR*-F1501**	F: ATCGATGAATTCTTAAGATATATCAATCCA	EcoRI	1500-1518
***LncHIFCAR*-R2166**	R: ATCGATGAATTCTTTTTGACAAAGGTCACAAA	EcoRI	2134-2153
***RMRP*-F**	F: AAATTTGAATTCGGTTCGTGCTGAAGGCCTGTATCCTAGGCT	EcoRI	1-30
***RMRP*-R**	R: TTTAAAGAATTCACAGCCGCGCTGAGAATGAGCCCCGTGT	EcoRI	241-271
***LncHIFCAR*-sh589-607 (Top)**	F: ATCGATGAATTCTTAAGATATATCAATCCA	EcoRI	589-607
***LncHIFCAR*-sh589-607 (Bottom)**	R: ATCGATGAATTCTTTTTGACAAAGGTCACAAA	EcoRI	589-607
***HIF1A*-F**	F: ATCGATCGGTCCGATGGAGGGCGCCGGCGGCGCGAAC		1-24
***HIF1A*-R**	R: ATCGATCGGACCGTCAGTTAACTTGATCCAAAGC		2461-2479
***HIF1A*-F1**	F: ATCGGGATCCATGGAGGGCGCCGGCGGC	BamHI	1-28
***HIF1A*-R80**	R: ATCGGCGGCCGCTCAATATCCAAATCACCAGC	NotI	49-80
***HIF1A*-F81**	F: ATCGGGATCCGAAGATGACATGAAAGCA	BamHI	81-108
***HIF1A*-R200**	R: ATCGGCGGCCGCTCTACGTGAATGTGGCCTGT	NotI	169-200
***HIF1A*-F201**	F: ATCGATGGATCCTATGATACCAACAGTAACCAACCT	BamHI	201-232
***HIF1A*-R329**	R: ATCGATGCGGCCGCTCATTCTTGGTGTTATATATGAC	NotI	298-329
***HIF1A*-F330**	F: ATCGGGATCCTCTCAACCACAGTGCATT	BamHI	330-357
***HIF1A*-R530**	R: ATCGGCGGCCGCTCTTCATTGACCATATCACT	NotI	499-530
***HIF1A*-F531**	F: ATCGGGATCCTTCAAGTTGGAATTGGTA	BamHI	531-555
***HIF1A*-R826**	R: ATCGGCGGCCGCTCGTTAACTTGATCCAAAGC	NotI	795-826

F, Forward; R, Reverse; RE, designed restriction enzyme cloning site.

*Based on human *LncHIFCAR* (MIR31HG; GenBank accession no. NR_027054), *RMRP* (GenBank accession no. NR_003051), and *HIF1A* (GenBank accession no. NM_001243084.1).

Supplementary Table 4. Probe sequences used in the ChIRP

Probe name	Sequence (5' to 3')
LncHIFCAR	
**ChIRP *LncHIFCAR*-1**	TCCGAGTAGGAGGACAGAAG
**ChIRP *LncHIFCAR*-2**	TTGTGTCCACAACACATTCT
**ChIRP *LncHIFCAR*-3**	TATTTCCAGGAATCCATCTC
**ChIRP *LncHIFCAR*-4**	ACACTTTACGTCATCAGCAG
**ChIRP *LncHIFCAR*-5**	TCTTTCCTCTATGATGTGTT
**ChIRP *LncHIFCAR*-6**	CCTTCTTGTGTCTAAAGGAC
**ChIRP *LncHIFCAR*-7**	TAGGATATAACCTGCCTCAG
**ChIRP *LncHIFCAR*-8**	GCCAAAAGCATCCTGATTTC
**ChIRP *LncHIFCAR*-9**	CTCCATTAAAGCCATGCATA
**ChIRP *LncHIFCAR*-10**	CCTCCTTTTAGGTCATATAG
**ChIRP *LncHIFCAR*-11**	GTTTCTCATCTGATTGATCA
**ChIRP *LncHIFCAR*-12**	GATCCTGATTTCCTATGCAA
**ChIRP *LncHIFCAR*-13**	CCATCAACGTCTTCTGTGAA
**ChIRP *LncHIFCAR*-14**	CCAGGGAAGCATAACCACAT
**ChIRP *LncHIFCAR*-15**	TTTCTTTTAGGGGTATTGGC
**ChIRP *LncHIFCAR*-16**	GTGAATCATCACTGCTGAGG
**ChIRP *LncHIFCAR*-17**	AAGAAGCAAGAACCTCCCTG
lacZ	
**ChIRP *lacZ*-1**	TAGCCAGCTTTCATCAACAT
**ChIRP *lacZ*-2**	AGCAGCAGACCATTTTCAAT
**ChIRP *lacZ*-3**	GTGTGGGCCATAATTCAATT
**ChIRP *lacZ*-4**	CGGCAGCCGTTATTATTATT
**ChIRP *lacZ*-5**	GAAACTGTTACCCGTAGGTA
**ChIRP *lacZ*-6**	CACGGCGTTAAAGTTGTTCT
**ChIRP *lacZ*-7**	GGATCGACAGATTTGATCCA
**ChIRP *lacZ*-8**	GTAGTTCAGGCAGTTCAATC
**ChIRP *lacZ*-9**	CAACGGTAATCGCCATTTGA
**ChIRP *lacZ*-10**	TGCAAGGCGATTAAGTTGGG
